# Suppression of autoimmune demyelinating disease by preferential stimulation of CNS-specific CD8 T cells using Listeria-encoded neuroantigen

**DOI:** 10.1038/s41598-017-01771-8

**Published:** 2017-05-08

**Authors:** Farah R. Itani, Sushmita Sinha, Ashley A. Brate, Lecia L. Pewe, Katherine N. Gibson-Corley, John T. Harty, Nitin J. Karandikar

**Affiliations:** 1 Department of Pathology, University of Iowa, 200 Hawkins Dr., Iowa City, IA 52242 USA; 2 Department of Microbiology, University of Iowa, 200 Hawkins Dr., Iowa City, IA 52242 USA; 3 Interdisciplinary Graduate Program in Immunology, University of Iowa, 200 Hawkins Dr., Iowa City, IA 52242 USA

## Abstract

CD8 T-cells predominate in CNS lesions of MS patients and display oligoclonal expansion. However, the role of myelin-specific CD8 T-cells in disease remains unclear, with studies showing protective and pathogenic roles in EAE. We demonstrated a disease-suppressive function for CNS-specific CD8 T-cells in a model where the antigen is exogenously administered *in vivo* and used for *in vitro* activation. To probe the nature of the CD8 response elicited by endogenously presented myelin antigens *in vivo*, we developed a novel approach utilizing infection with *Listeria monocytogenes* (LM) encoding proteolipid protein peptide (PLP) amino acids 178-191 (LM-PLP). LM-PLP infection preferentially induced PLP-specific CD8 T-cell responses. Despite the induction of PLP-specific CD8 T-cells, LM-PLP infection did not result in disease. In fact, LM-PLP infection resulted in significant amelioration of PLP178-191-induced EAE. Disease suppression was not observed in mice deficient in CD8 T-cells, IFN-γ or perforin. DTH responses and CNS infiltration were reduced in protected mice, and their CD4 T-cells had reduced capacity to induce tissue inflammation. Importantly, infection with LM-PLP ameliorated established disease. Our studies indicate that CD8 T-cells induced by endogenous presentation of PLP178-191 attenuate CNS autoimmunity in models of EAE, implicating the potential of this approach as a novel immunotherapeutic strategy.

## Introduction

Multiple sclerosis (MS), the most common neurologic disease of young adults, is a T cell-mediated demyelinating disease of the central nervous system (CNS) in which autoreactivity results in progressive impairment in neurologic function^1^. Within MS lesions, CD8 T cells show evidence of oligoclonal expansion, indicative of an important yet unidentified functional role in disease^2^. Studies of the immune basis of MS or its animal model, experimental autoimmune encephalomyelitis (EAE), have largely been directed toward the study of Th1 and Th17 effector CD4 T cells mediating pathology, while fewer studies have addressed the involvement of CD8 T cells in disease development and regulation.

Different subsets of CD8 T cells have been described as pathogenic effectors and/or regulators of the immune response in EAE. Studies have utilized both myelin-targeted and non-myelin antigen-driven systems to examine the pathogenic potential of CD8 T cells (reviewed in refs 3, 4). The majority of these studies capitalize on the genetic manipulation of mice, while few depict the involvement of myelin-specific CD8 T cells in the pathogenesis of CNS disease in a wild-type setting^5–9^. Conversely, other studies, including those from our laboratory, have demonstrated a protective role for CD8 T cells in both EAE and MS^10, 11^.

Studies in human MS have shown that CNS-specific CD8 T cells are regulatory in nature^10, 11^. Of note, CD8 T cell suppressive function is dampened during acute disease exacerbation but restored during remission, underscoring the clinical importance of this function^12^. Furthermore, these regulatory cells were found to be contained within the terminally differentiated CD8 T cell pool, and this subset was lacking during disease exacerbation^13^. We have been able to model this disease regulatory role of CD8 T cells in EAE models. We have observed that myelin-specific CD8 T cells are autoregulatory in nature and suppress disease by affecting encephalitogenic CD4 T cell and modulating dendritic cell (DC) function^10, 11, 14^.

In particular, we have shown disease suppressive function in myelin oligodendrocyte glycoprotein (MOG) peptide 35–55-induced CD8 T cells in the B6 model as well as PLP178-191-induced CD8 T cells in both B6 and SJL mice^10, 11, 14^. However, in these systems the response-inducing antigen was administered exogenously in the form of a CFA-containing immunization (which is the standard protocol for EAE induction) or the addition of these peptides to *in vitro* cultures. Thus, induction of CD8 T cell responses would involve processing and cross-presentation of the antigens^15^.

In the current study, we developed a novel system whereby a myelin antigen would be presented endogenously *in vivo* through the routine Class I pathway and asked whether these *in vivo*-generated CD8 T cell responses were pathogenic or regulatory in nature. Thus, we genetically engineered the intracellular bacterium *Listeria monocytogenes* (LM), a pathogen commonly used to induce and characterize CD8 T cell responses^16^, to express PLP antigen. Herein we report that myelin-specific CD8 T cells generated through such endogenous processing and presentation of CNS antigen are disease regulatory in nature, implicating a novel therapeutic strategy for this disease.

## Results

### Infection with an attenuated strain of *L. monocytogenes* encoding for PLP-178-191 generates CNS-specific CD8 T cells, with no evidence of pathogenicity

In prior studies, we have observed suppression of EAE by MOG35-55- and PLP178-191-specific CD8 T cells. Suppression by PLP178-191-specfic CD8 T cells was not only more robust than MOG35-55 in the B6 model, but PLP178-191-specfic CD8 T cells were also suppressive in SJL mice^10^. In all these systems, antigen was administered exogenously as a CFA-based immunization and used *in vitro* for CD8 T cell stimulation, depending on cross-presentation in MHC Class I. To evaluate the role of myelin-specific CD8 T cells when induced through *in vivo* endogenous antigen presentation, we decided to genetically engineer an attenuated strain of *L. monocytogenes* to express a sequence encoding PLP178-191 (LM-PLP), using a previously characterized vector system (Fig. S1)^17^. As an antigen-delivery system, attenuated *L. monocytogenes* establishes a self-limited, natural infection within the host leading to the secretion and endogenous presentation of encoded antigens, with induction of MHC Class I-restricted CD8 T cell responses^18^ and can be safely administered to interferon-gamma (IFN-γ) or perforin-deficient mice^19^, while maintaining the ability to induce CD8 T cell responses.

**Figure 1:**
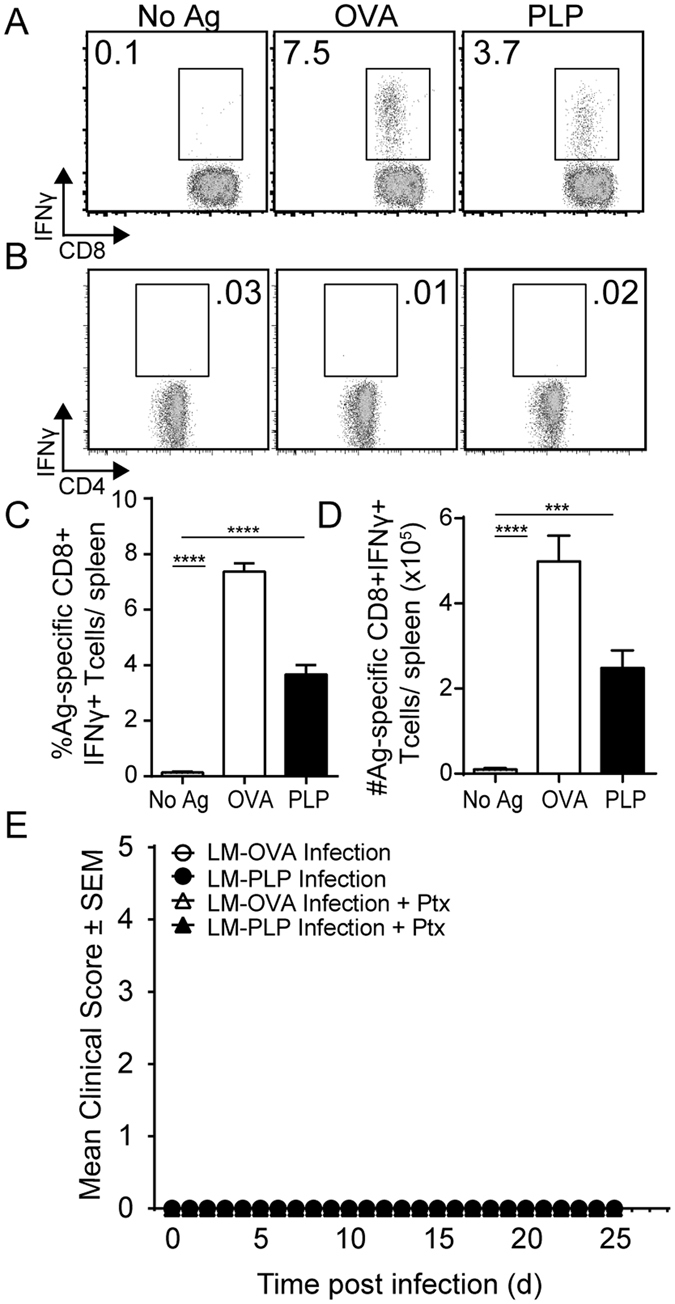
Infection with LM-PLP generates PLP-specific CD8 T cells that are not pathogenic. Intracellular cytokine staining was performed using bulk splenocytes harvested from LM-PLP infected B6 mice on d7 post infection. Splenocytes were left unstimulated, or stimulated with either OVA257-264 (positive control) or PLP178-191. Representative flow plots show CD8 (**A**) and CD4 (**B**) T cell responses from LM-PLP-infected mice. Summary bar graphs display the frequency (**C**) and absolute numbers (**D**) of antigen-specific CD8 T cell responses to indicated antigens. (**E**) Mice were infected with either LM-OVA or LM-PLP and followed for neurologic signs of disease with or without pertussis toxin administration. Data are representative of at least three independent experiments in each panel with at least six mice per group per experiment. ***p < 0.001, ****p < 0.0001.

We first asked whether infection with LM-PLP would induce CD8 T cell responses. Using standard intracellular cytokine staining, we observed that LM-PLP infection induced PLP-specific CD8 T cells, detected by IFN-γ production 7 days post infection (Fig. 1A). The LM-PLP vector also contained the OVA sequence backbone as an internal control (Fig. S1); therefore, OVA-specific CD8 T cell responses were detected in both LM-PLP-infected mice (Fig. 1A) as well as LM-OVA-infected mice (data not shown). Of note, LM-PLP infection did not result in a detectable PLP178-191-specific CD4 T cell response (Fig. 1B). Moreover, we also could not detect CD4 or CD8 T cell responses to unrelated myelin peptides MOG35-55 or PLP139-151 (data not shown), indicating the antigenic specificity of the detected response. Frequencies of autoantigen-specific cells ranged between 2-5% of CD8 T cells, which were lower than frequencies of OVA-specific CD8 T cell responses and similar to what has been previously reported using other self-antigen LM infection systems (Fig. 1C)^20^. Absolute numbers of PLP-specific CD8 T cells in LM-PLP infected mice averaged 2.4 × 10^5^ cells/spleen (Fig. 1D). A dose response experiment revealed that optimal PLP-specific CD8 T cell IFN-γ responses were detected at 100 μM of self-peptide (Fig. S2), which is significantly higher than the concentration of OVA peptide used (2 μM). Again, no CD4 T cell responses were detected in these cultures (Fig. S2), suggesting the specific stimulation of CD8 T cells following infection.

To ask whether these induced PLP-CD8 T cell responses may be capable of inducing CNS disease; we observed LM-OVA- and LM-PLP-infected mice for over 40 days either following infection alone or after injecting pertussis toxin. None of the groups developed clinical signs of neurologic disease (Fig. 1E), signifying that these responses were not sufficient to cause CNS pathology on their own.

### LM-PLP infection leads to amelioration of EAE

**Figure 2:**
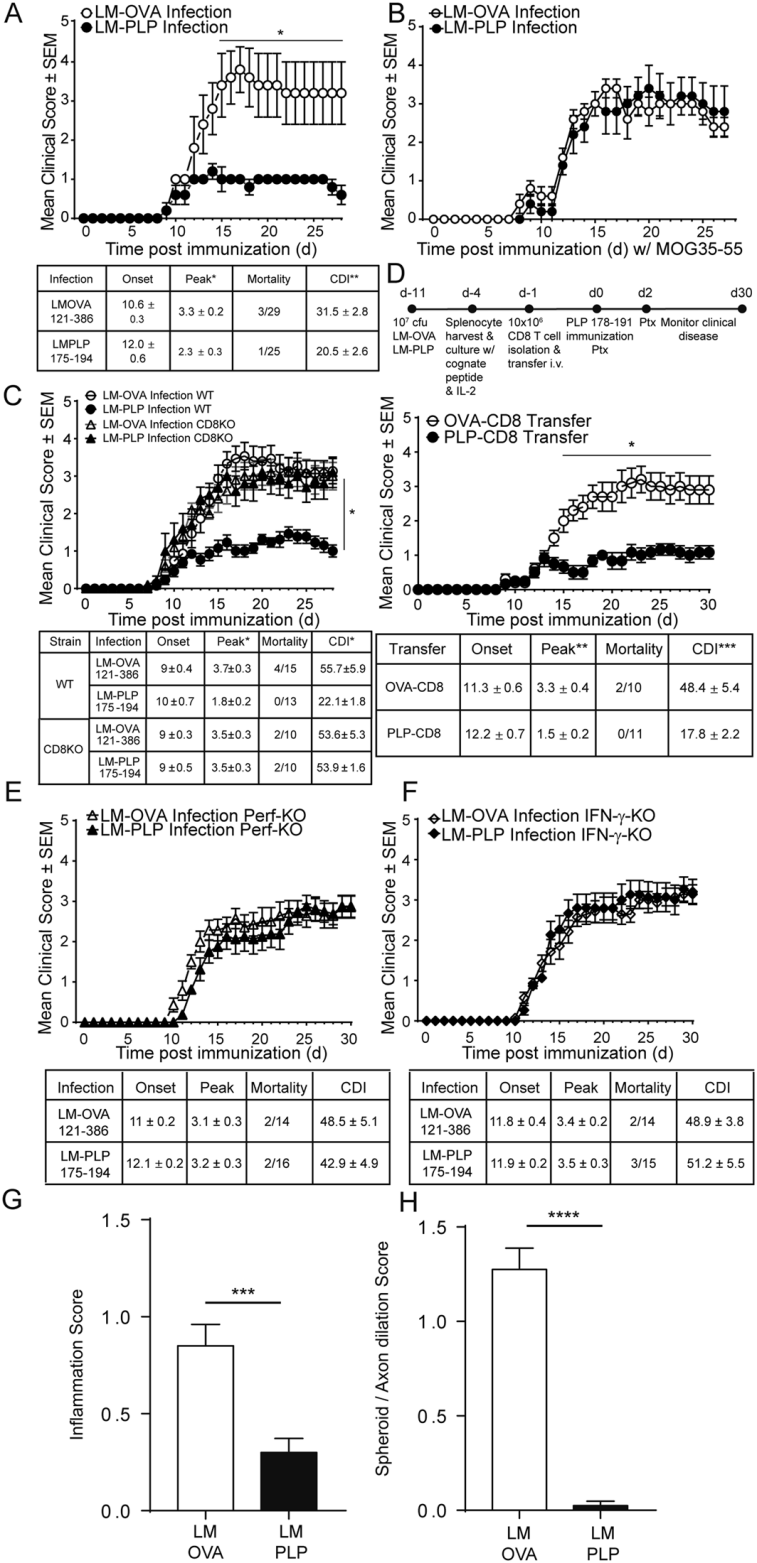
LM-PLP infection protects mice from EAE in a CD8-dependent antigen-specific manner. (**A**) B6 mice were infected with LM-PLP or LM-OVA on d-7 and immunized with PLP178-191/CFA on d0 and monitored for clinical disease. (**B**) B6 mice were infected with LM-PLP or LM-OVA, followed by induction of EAE with non-cognate antigen MOG35-55/CFA. Disease scores are shown. (**C**) CD8-KO mice were infected with either LM-PLP or LM-OVA on d-7, immunized with PLP178-191/CFA on d0 and monitored for clinical disease. (**D**) CD8 T cells harvested from LM-PLP or LM-OVA-infected B6 mice were stimulated *in vitro* with PLP178-191 and OVA257-264 respectively, and adoptively transferred into naïve recipients followed by subsequent immunization with PLP178-191/CFA and the monitoring of clinical disease. (**E**) Perf-KO mice were infected with LM-PLP or LM-OVA on d-7, immunized with PLP178-191/CFA on d0, and monitored for clinical disease. (**F**) IFN-γ-KO mice were infected with LM-PLP or LM-OVA on d-7, immunized with PLP178-191/CFA on d0, and monitored for clinical disease as depicted. (**G**) Inflammation and (**H**) Spheroid/axon dilation scores from H&E stained lumbar spinal cord sections from infected and immunized mice on d18 post-immunization. Data are representative of at least two independent experiments in each panel. *p < 0.05, **p < 0.01, ***p < 0.001 ****p < 0.0001.

We next tested whether the PLP-specific CD8 T cells generated *in vivo* following infection could influence the clinical course of EAE. On day 7 post-infection, EAE was induced by immunization with PLP178-191/CFA and clinical signs of EAE were monitored. Compared to the LM-OVA control group, mice that were infected with LM-PLP exhibited attenuated clinical disease, with significantly reduced peak disease scores and cumulative disease indices (CDI); LM-OVA vs. LM-PLP: peak disease 3.3 ± 0.2 vs. 2.3 ± 0.3, p < 0.05; CDI 31.5 ± 2.8 vs. 20.5 ± 2.6, p < 0.01 (Fig. 2A). To elucidate whether this disease attenuation was antigen specific, we induced EAE in the LM-PLP- and LM-OVA-infected groups using MOG35-55/CFA immunization. In contrast to what was observed with PLP178-191/CFA immunization, MOG35-55/CFA-immunized animals were not protected from disease, implicating the importance of antigenic specificity for disease attenuation (Fig. 2B). The PLP178-191 peptide has also been defined in a separate model of EAE, and is used to induce a relapsing-remitting course of disease in SJL/J mice. Infection of SJL/J mice with LM-PLP followed by PLP178-191/CFA immunization led to the suppression of clinical disease similar to what was observed in the B6 model (Fig. S3), suggesting that PLP-specific CD8 T cells generated following infection can regulate disease in two different models of EAE.

### CD8 T cells are required for LM-PLP-induced disease protection

We observed that LM-PLP infection resulted in the induction of CD8 T cell responses and led to disease attenuation. Thus, we wanted to directly test the hypothesis that CD8 T cells are required to mediate this disease suppression. Mice deficient for CD8α (CD8-KO) were infected with either LM-OVA or LM-PLP, followed by immunization with PLP178-191/CFA at day 7. Interestingly, CD8-KO mice failed to regulate EAE when infected with LM-PLP, unlike their WT counterparts (Fig. 2C). Peak disease and CDI did not differ between immunized CD8-KO mice infected with either LM-OVA or LM-PLP (peak scores: 3.5 ± 0.3 vs. 3.5 ± 0.3; CDI: 53.6 ± 5.3 vs. 53.9 ± 1.6). These observations demonstrate the importance of myelin-specific CD8 T cells in down-modulating clinical EAE.

We further tested whether CD8 T cells isolated from infected mice were sufficient to attenuate EAE in recipient animals. Therefore, we obtained CD8 T cells from infected mice at day 7 post-infection, stimulated them *in vitro* with either OVA or PLP peptides and adoptively transferred these into naïve recipients, followed by induction of EAE with PLP178-191/CFA immunization. Mice that received CD8 T cells from LM-PLP infected donors exhibited significantly reduced peak disease scores (1.5 ± 0.2 vs. 3.3 ± 0.4, p < 0.01) and CDI (17.8 ± 2.2 vs. 48.4 ± 5.4, p < 0.001), in comparison to LM-OVA controls (Fig. 2D). Our data show that infection with LM-PLP alone can generate peptide-specific CD8 T cells that are sufficient to transfer disease protection.

We have previously demonstrated that disease suppression by CNS-specific CD8 T cells is dependent on expression of IFN-γ and perforin by the CD8 T cells^11^. IFN-γ-producing CD8 T cells with disease-suppressive function have also been described by several other groups^21–23^. To test whether these effector molecules are important in infection-induced autoregulatory CD8 T cells, we infected perforin- or IFN-γ-KO animals with either LM-OVA or LM-PLP and induced EAE through PLP178-191/CFA immunization. Experimental and control KO groups did not differ in terms of disease course, onset, peak, mortality, or CDI (Fig. 2E and F). IFN-γ- and perforin-KO animals failed to attenuate disease when infected with LM-PLP, suggesting that the endogenously induced CNS-specific CD8 T cells might mediate a cytotoxic regulatory mechanism during disease attenuation.

Finally, histopathologic analysis and semi-quantitative scoring revealed significant differences between the two groups, with less multifocal inflammation and axonal swelling observed in LM-PLP-infected animals when compared to LM-OVA counterparts (Figs S4, 2G,H).

### Cell-mediated immune function is dampened following LM-PLP infection

**Figure 3:**
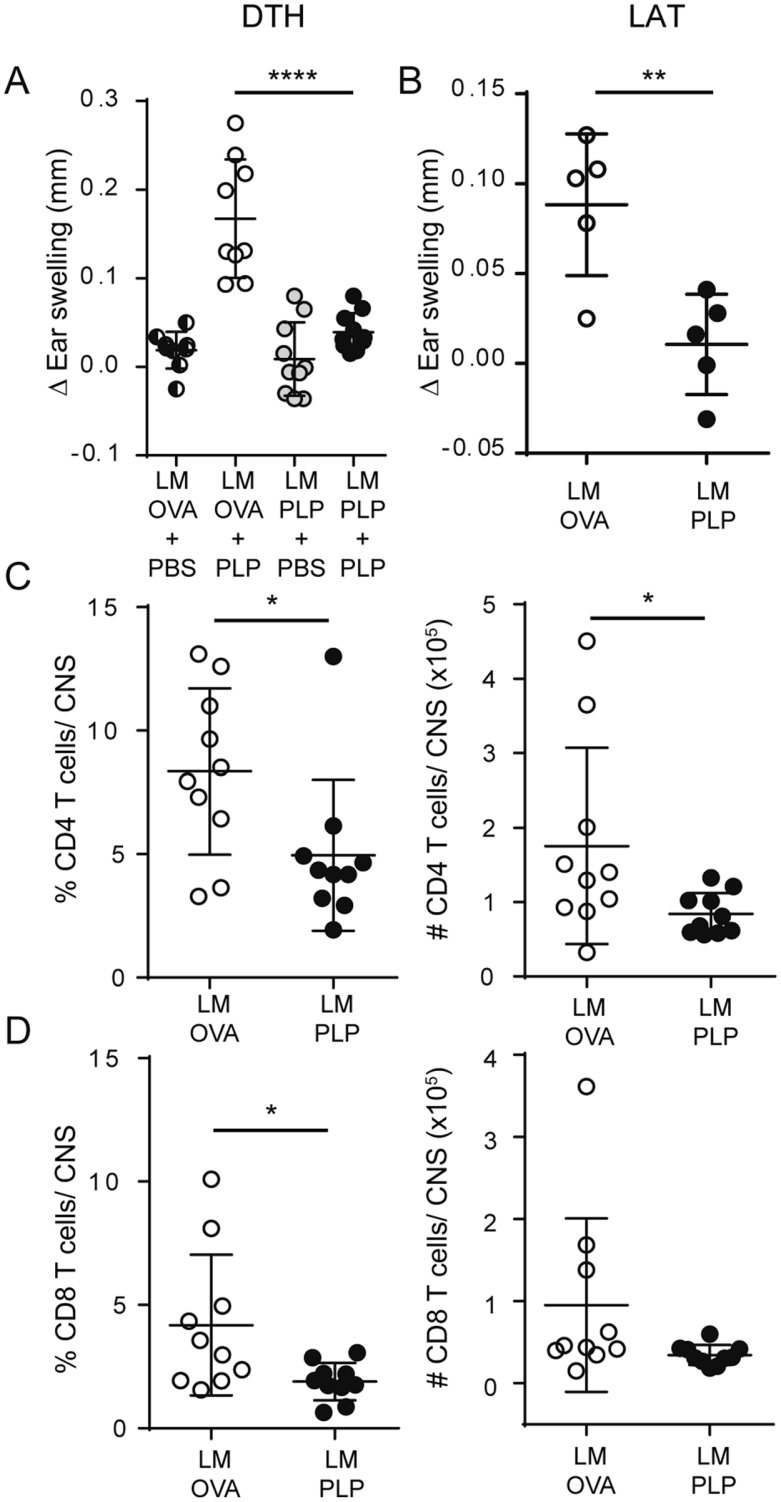
LM-PLP infected mice have reduced cell-mediated immunity and CNS cellular infiltration and demyelination. (**A**) B6 mice infected with LM-OVA or LM-PLP on d-7 and immunized with PLP178-191 on d0 were challenged intradermally on the ear d10 post-immunization using PLP178-191 antigen or PBS control. Change in ear swelling was measured 48 h later. (**B**) CD4 T cells were harvested from LM-PLP or LM-OVA-infected and PLP-immunized mice and transferred into the ears of naïve B6 mice along with APCs and cognate antigen. Ear swelling was measured 48 h later. Mice were infected with LM-PLP or LM-OVA on d-7 and immunized with PLP178-191/CFA on d0. CNS tissues were processed for flow cytometric analysis on d10 post immunization. Frequencies and absolute numbers of CD4 (**C**) and CD8 (**D**) T cells are shown; populations are gated through CD45 + Thy1.2 + cells. Data are representative of at least three independent experiments. *p < 0.05, **p < 0.01, ***p < 0.001.

To further dissect the mechanism of LM-PLP-mediated disease suppression, we tested cell-mediated immune function using the delayed-type hypersensitivity (DTH) reaction, in which the inflammatory response is known to be mediated predominantly by CD4 T (Th1) cells^24, 25^. LM-OVA and LM-PLP-infected animals were challenged on the ears with PLP178-191 peptide at day 10 post-PLP178-191/CFA immunization (17 days post infection) and the ensuing inflammatory response was measured by change in ear thickness. In comparison to control animals, the specific DTH response in LM-PLP infected mice was significantly reduced (mean Δ ear swelling ± SEM: 0.039 ± 0.006 vs. 0.167 ± 0.022; p < 0.0001), indicative of a dampened Th1 response to PLP (Fig. 3A). To directly test whether CD4 T cells from the two groups had different inflammatory ability, we used a local adoptive transfer (LAT) assay system, which can measure the ability of antigen-specific CD4 T cells to cause ear swelling post-antigenic stimulation^26^. Thus, we transferred CD4 T cells harvested from the spleens of LM-PLP or LM-OVA-infected and PLP178-191-immunized animals along with irradiated splenocytes (as APCs) and PLP peptide (as antigen) into the ears of naïve mice. Ear swelling was measured at 24 hours and showed that CD4 T cells from LM-PLP-infected animals were significantly inhibited in their ability to cause ear swelling in comparison to controls (mean Δ ear swelling ± SEM: 0.011 ± 0.012 vs. 0.088 ± 0.018; p < 0.01) (Fig. 3B).

We next evaluated the CNS infiltration and demyelination, using flow cytometry and CNS histopathology. Consistent with the observation that LM-PLP infected animals develop attenuated clinical disease and have dampened cell mediated immunity, we observed decreased frequencies of CD4 and CD8 T cells in the CNS of LM-PLP-infected animals when compared to controls (Fig. 3C,D).

### Infection with LM-PLP treats already established disease

**Figure 4:**
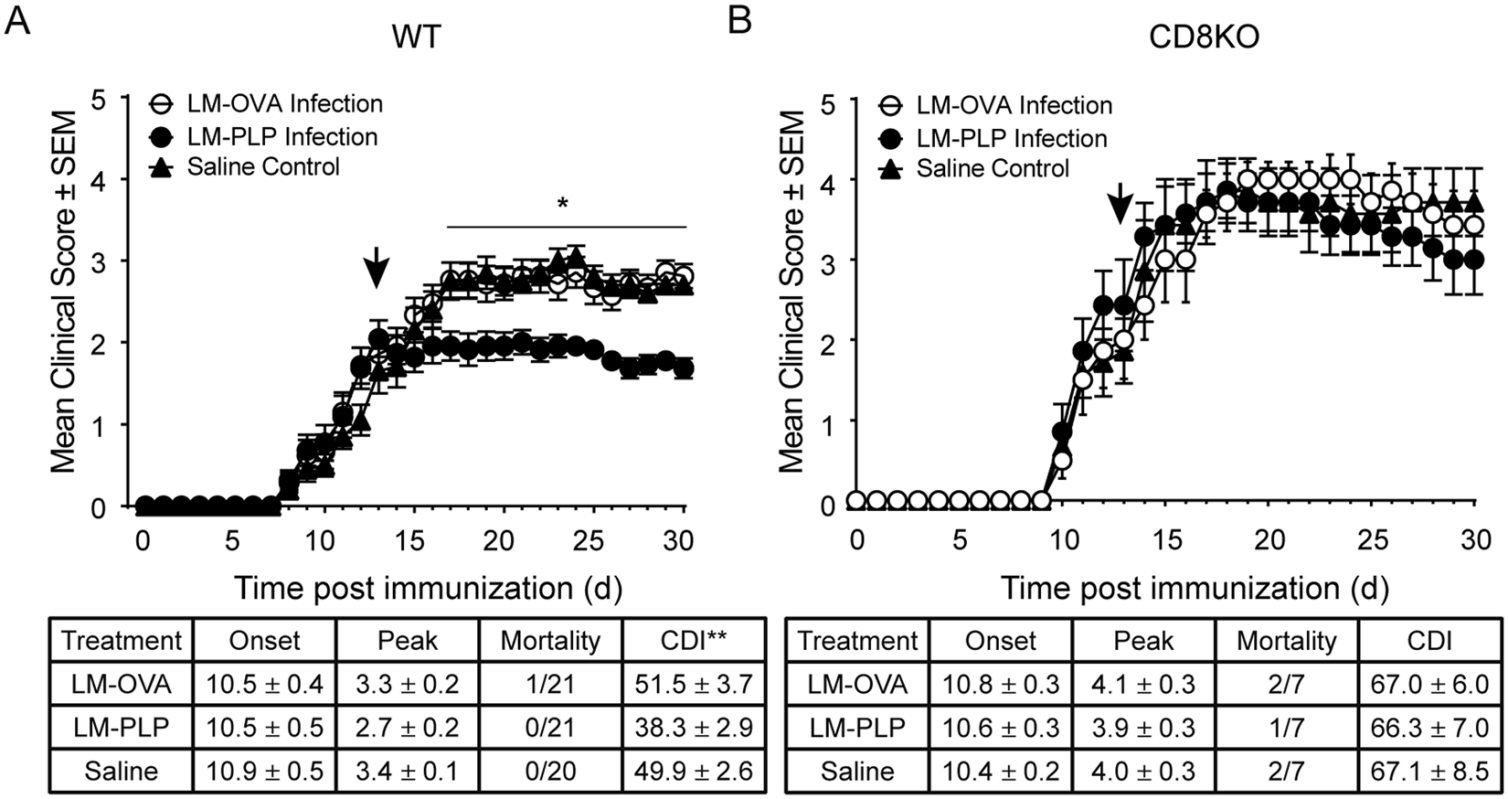
Infection with LM-PLP attenuates already established disease in a CD8-dependent manner. (**A**) Wild-type B6 or (**B**) CD8KO mice were immunized with PLP178-191/CFA and administered pertussis toxin on d0 and d2. Mice were monitored for clinical disease and were infected with LM-PLP or LM-OVA or injected with saline on d13 post-immunization. Data are representative of two independent experiments. *p < 0.05, **p < 0.01, ***p < 0.001.

Since LM-PLP infection prior to disease induction was protective, we decided to test the therapeutic potential of this approach. Mice were immunized with PLP178-191/CFA and monitored for clinical disease, and on day 13 post-immunization (during the acute phase of disease), animals were infected with either LM-OVA, LM-PLP, or administered saline. Animals infected with LM-PLP exhibited reduced clinical disease scores when compared to the other treatment groups, as evidenced by their reduced CDI (38.3 ± 2.9 vs. 51.5 ± 3.7, 49.9 ± 2.6; p < 0.01) (Fig. 4A). To establish the importance of CD8 T cells in disease amelioration, we conducted similar experiments using CD8-KO mice. CD8-KO animals infected with LM-PLP after established EAE exhibited similar clinical disease to those infected with LM-OVA or given saline with comparable disease onset, peak, mortality, and CDI parameters (Fig. 4B), indicating that LM-PLP treatment of ongoing EAE was also mediated through the induction of autoregulatory CD8 T cell responses.

## Discussion

The function of CD8 T cells in MS is poorly understood and is of particular interest, as they outnumber CD4 T cells in CNS lesions of human MS and exhibit oligoclonal expansion^2, 27^. Contradictory roles for CNS-specific CD8 T cells in the development and/or regulation EAE have been implicated by several different studies^7–9, 22, 28^. Almost all of these studies either used transgenic models with CNS-expressed foreign antigens as the target or employed immunization techniques that depended on cross-presentation for the induction of CD8 T cell responses. In this study, we have addressed for the first time the role of endogenously primed CNS-specific CD8 T cells, by developing a model of *in vivo* infection. We demonstrate that *in vivo* priming of CNS-specific CD8 T cells does not lead to neurologic disease. In fact, LM-PLP infection results in preferential priming of a CNS-specific CD8 T cell response, which downregulates EAE in an antigen specific manner and is able to do so even after disease is established. We believe that these findings open a novel immunotherapeutic strategy for this disease.

Our prior studies have pointed to a regulatory role for myelin-specific CD8 T cells in active and adoptive forms of EAE^10, 11, 14^. In these studies, there was co-induction of CD4 and CD8 T cell responses through exogenous antigenic priming and the regulation of disease by CD8 T cells was mediated by their targeting of encephalitogenic CD4 T cells and modulation of DC function^10, 11^. An unresolved issue in these studies was whether CD8 T cells induced through *in vivo* presentation of myelin antigen would have similar functional roles as those induced through exogenous priming and cross-presentation. To address this issue, we used an approach to preferentially target myelin antigens to CD8 T cells endogenously by utilizing infection with recombinant *Listeria monocytogenes* encoding myelin proteolipid protein PLP178-191. A large body of work characterizing mammalian immune-system pathogens has resulted from studying this facultative intracellular bacterium^29^. Multiple *Listeria* strains have been engineered and subsequently used to probe heterologous antigen-specific responses. These include recombinant strains expressing lymphocytic choriomeningitis virus (LCMV) and *Plasmodium yoelii*, to name a few^30–32^. In these studies, LM infection primed antigen-specific CD8 T cells to recognize MHC-class I-restricted epitopes of interest. For the most part, *L. monocytogenes* infection has been used in the context of infectious diseases to study the CD8 T cell response. There have been few studies that have used this system to generate anti-self CD8 T cell responses against melanoma antigens such as TRP-2, which led to protection from B16 melanoma challenge^32^. Here we have exploited this system, for the first time, to generate a myelin-specific CD8 T cell response.

Historically, CD8 T cell responses to self-antigen are difficult to detect and require a stronger peptide stimulus than foreign antigen responses^9^. Interestingly, infection of B6 mice with our newly generated LM-PLP strain preferentially primed antigen-specific CD8 T cell responses. As depicted by our peptide dose-experiments, while PLP178-191 specific CD8 T cell response were detected with peptide concentrations as low as 20 μM, maximal response was detected when 100 μM of self-antigen was used for stimulatory conditions.

Despite the induction of PLP-specific CD8 T cells, infection alone with LM-PLP did not result in neurologic disease, even with pertussis toxin administration. This corroborates our previously published observations that CNS-specific CD8 T cells derived from mice with EAE could not transfer disease to naïve mice^11, 14^ nor could they be detected in the CNS unless active EAE was induced in the recipient mice^11^. Our current data from the infection model argue against an independently pathogenic role for these autoreactive CD8 T cells.

In fact, preferential pre-activation of PLP-specific CD8 T cells by LM-PLP infection led to the significant amelioration of clinical disease induced by routine PLP178-191/CFA immunization. This amelioration was antigen-specific and was seen in both B6 and SJL mice. CD8 T cells were necessary to see this effect and CD8 T cells from infected animals could transfer protection to new recipients. These findings indicate that endogenously primed autoreactive CD8 T cells that are induced through an infection, without dependence on peptide cross-presentation or concomitant CD4 T cell responses, are autoregulatory in nature. In that regard, their biologic behavior in disease is similar to the CD8 T cell response primed by immunization with encephalitogenic peptide^10, 11, 14^. Taken together, both systems provide strong evidence that CNS-specific CD8 T cells are important mediators of disease regulation/protection. In a resurgence of the concept of the “suppressor” CD8 T cell (at a functional level), similar disease inhibitory properties have been described for CD8 T cells in other autoimmune diseases including inflammatory bowel disease, type 1 diabetes, and rheumatoid arthritis^33–35^.

Infection-induced CD8 T cells were dependent on IFN-γ and perforin production to mediate their protective effects, suggesting a cytotoxic/regulatory mechanism for suppression. Anti-inflammatory effects of IFN-γ are increasingly being recognized. IFN-γ has been shown to induce IDO production by DCs, thereby leading to the inhibition of T cell priming^36^. We have also shown that regulatory CD8 T cells require an intact IDO pathway and modulate splenic DCs^10^. It has been shown that exposure to IFN-γ *in vitro* renders splenic DCs immature and of a tolerogenic phenotype, and when transferred, these cells decrease disease severity in multiple EAE models^37^. Neutrophils and myeloid-derived suppressor cells (MDSCs) have also been shown to inhibit T cell proliferation in EAE through IFN-γ dependent mechanisms^38–40^. Overall in LM-PLP-infected mice, we observed a dampened inflammatory response following ear challenge with antigen. Furthermore, CD4 T cells derived from protected mice showed reduced ability to induce ear swelling in LAT assays. Absence of disease suppression in the perforin-KO mice suggests that target cell killing is an important mechanism used by CD8 T cells for disease amelioration. However, the specific target population is still unclear. Based on previously published studies^11, 14^ and some ongoing unpublished observations, we hypothesize that CD8 T cells may be directly killing pathogenic CD4 T cells (and potentially APC subsets) in our model. We propose that CNS antigen may find its way into MHC Class I complexes on CD4 T cells through trogocytosis in an environment where APC, CD4 and CD8 T cells are in close interaction. We are addressing these hypotheses in ongoing studies.

Our studies demonstrate two potential immunotherapeutic strategies for this disease. One strategy is the transfer of *in vitro*-activated CNS-specific autoregulatory CD8 T cells, derived from either immunized or infected mice. In human MS, we have shown that exacerbation is associated with the lack of CD8 T cell regulatory function, which is restored in the quiescence phase of MS^13^, suggesting that maintenance of CD8 T cell regulatory function through transfer of autologous CD8 T cells may be an effective way of preventing relapses in MS. However, cellular adoptive therapies suffer from inherent limitations in that they are time-consuming, costly, and of limited success, making this approach less attractive in the clinic.

We now show that LM-PLP infection not only protects mice from EAE but also ameliorates already established disease. This generates the second possible strategy for disease treatment – i.e., LM-PLP infection as a vaccination strategy for MS. Previous studies have demonstrated the safety of live attenuated double-deficient (LADD) LM vaccines that stimulate both the innate and adaptive immune systems in human clinical trials^41^. This provides a proof-of-principle that *in vivo* stimulation of autoreactive CD8 T cells through a genetically engineered infectious agent may be a promising avenue for disease treatment. We will address this hypothesis in upcoming preclinical studies.

In summary, we make the observation that targeting myelin peptide presentation to CD8 T cells through an infection/vaccination attenuates CNS autoimmunity. We believe that these findings may help generate a novel future therapeutic strategy for this and other immune-mediated diseases.

## Materials and Methods

### Mice

All experiments used female six to eight-week-old mice that were housed in climate-controlled facilities under the supervision of certified veterinarians, maintained on twelve hour lights on/off cycle, and allowed food and water ad libitum. Wild-type (WT) C57BL/6, SJL/J, CD8 knock-out (KO) *Cd8a* ^*tm1Mak*^, Interferon-γ KO B6.129S7-*Ifng* ^*tm1Ts*^/J, and Perforin KO C57BL/6-Prf1^tm1Sdz^/J mice were purchased from The Jackson Laboratory (Bar Harbor, ME) and housed in specific pathogen-free animal facilities. For infection studies, mice were transferred to biosafety level 2 conditions. All animal experiments were approved by the University of Iowa animal care and use committee protocols. All methods were performed in accordance with relevant guidelines and regulations.

### Generation of Recombinant Listeria-expressing Myelin T cell Epitopes

Attenuated (Δ*actA*/Δ*inlB*) recombinant *Listeria monocytogenes*–expressing proteolipid protein (PLP) T cell epitopes was generated as described previously^17, 18^. Briefly, we generated LM codon-optimized constructs containing amino acid coding sequences of the defined PLP178-191 sequence, with three flanking amino acids on each end to encourage natural processing. The following sequence was used: (IYFNTWTTCQSIAFPSKTSA). Integration of the desired sequence occurred between BamHI and PstI restriction sites of the vector (Fig. S1), and was confirmed by sequencing clones for the inserted epitopes. The control strain of LM contains an insert encoding for ovalbumin peptide 121–386 (LM-OVA) and the LM-PLP strain contains sequences encoding both OVA and PLP peptides, allowing for an internal control. Clones were transformed into conjugation donor cells and mated with an attenuated strain of LM with deleted *actA* and *inlB* genes. Recombinant *L. monocytogenes* strains were grown and prepared for injection as previously described^42^.

### *L. monocytogenes* infection, active EAE and Autoregulatory CD8 T cell Transfer

Mice were injected i.v. with 10^7^ cfu of recombinant LM. Seven days post-infection, these mice were immunized s.c. with emulsion containing Complete Freud’s Adjuvant supplemented with 200 μg *Mycobacterium tuberculosis* H37Ra (Difco, Detroit, MI) and 50–100 μg PLP_178–191_ (ChinaPeptides) distributed over two sites on the flank. In all models, 250 ng pertussis toxin was administered i.p. on days 0 and 2 post-immunization. For CD8 T cell transfer, splenocytes were collected on day seven post- LM infection and reactivated with 20 μg/ml cognate antigen and 10 pg/ml recombinant human IL-2 (University of Iowa Hospitals and Clinics) for 72 h at 37 °C. CD8 T cells were magnetically isolated using Ly-2 microbeads (Miltenyi) and 10^7^ live cells were transferred to recipient mice i.v. After 24 h, active EAE was induced using PLP178-191/CFA immunization as described above. Clinical signs of EAE were assessed daily, and animals were scored based on previously defined criteria^10^.

### *Ex vivo* Peptide Stimulation and Intracellular Cytokine Staining

Splenocytes from LM infected mice were resuspended in RPMI 1640 supplemented with 10% FBS, 1% L-glutamine, 50 U/ml penicillin, 50 mg/ml streptomycin, and 50 mM 2-ME. Cells were stimulated with OVA257–264, PLP178-191 (1 mM–200 μM), or no peptide in the presence of GolgiStop (BD Biosciences). Cultures were incubated for 5 h at 37 °C. Cells were washed with staining buffer and incubated with cell surface marker antibodies. After washing, cells were resuspended in Cytofix/Cytoperm (BD Biosciences) for 15 min at room temperature (RT). Following fixation, cells were washed with Perm/Wash (BD Biosciences), stained with antibodies specific for CD4, CD8, CD90.2, IFN-γ, or TNF-α, and processed for flow cytometric analysis.

### Delayed-Type Hypersensitivity (DTH) and Local Adoptive Transfer (LAT) assays

DTH and LAT assays were performed as described elsewhere^43–46^. For DTH experiments, mice infected with LM-OVA or LM-PLP were immunized with PLP178-191/CFA 7 days post-infection. Ten days post-immunization, mice were challenged intradermally in the ear pinna with antigen or vehicle control. Ear thickness was measured 48 h later and swelling responses were calculated. For LAT experiments, mice infected with LM-OVA or LM-PLP were immunized with PLP178-191/CFA seven days post-infection, and at 10 days post-immunization, they were euthanized and spleens were harvested. CD4 T cells from both groups were isolated following magnetic bead sorting (Miltenyi Biotec) protocols. CD4 T cells combined with naïve irradiated splenocytes and PLP antigen were intradermally injected into the ear pinna of naïve mice. Ear swelling responses were measured 24 h and 48 h post injection. In both assays, ear thickness was measured using an engineer’s micrometer (Mitutoyo).

### Isolation of CNS Leukocytes and Flow Cytometric Analysis

Immune cells from CNS tissue were harvested from combined brain and spinal cords and isolated by Percoll gradient centrifugation. Mice were anesthetized and perfused through the left ventricle with 5% heparin in PBS solution. Spinal cords were obtained by flushing the vertebral canal with cold RPMI supplemented with 10% FBS (RP10) and brains were removed and placed in RP10. Tissues were forced through nylon mesh to yield a single cell suspension. Following Percoll gradient centrifugation, CNS leukocytes were collected from the interface, washed, and resuspended in staining buffer. CNS cells were stained with the indicated antibodies (BD and eBioscience), as described previously^11^.

### Histology and Tissue Scoring

Routine H&E staining was performed on formalin-fixed, paraffin embedded brain and spinal cord sections. Digital images were collected with a DP73 camera and CellSens software (Olympus, Tokyo, Japan). Stained spinal cord sections were evaluated for EAE lesions, including axonal sheath swelling, spheroid formation and inflammatory cell infiltration in a blinded manner, by a board-certified veterinary pathologist (K.N.G-C). The inflammation score was based on a scale from 0 to 3 where 0 = no inflammation; 1 = 1 to 4 small foci of low numbers of inflammatory cells; 2 = greater than 5 small foci or 1–3 larger accumulations of moderate to abundant numbers of inflammatory cells; 3 = greater than 5 large accumulations of moderate to abundant numbers of inflammatory cells. The spheroid/axonal dilation score was also based on a scale of 0–3 where 0 = no dilated axonal sheaths and/or spheroids present; 1 = rare, scattered and individualized empty dilated axonal sheaths and/or spheroids; 2 = 1 to 3 foci of small clusters of empty dilated axonal sheaths and/or spheroids; 3 = greater than 3 foci of small to large clusters of empty dilated axonal sheaths and/or spheroids.

### Statistical Analysis

Statistical analyses were performed using GraphPad Prism 7.0 (GraphPad Software, La Jolla, CA). Single comparisons of two means were analyzed by the Student *t* test. For multiparametric data, two-way ANOVA with Bonferroni post-test was used. For non-parametric data analysis (inflammation and axonal scores), the Mann-Whitney test was used. The *p* values < 0.05 were considered significant.

### Data Availability

The datasets generated and/or analyzed during the current study are either included in the manuscript or available from the corresponding author on reasonable request.

## Electronic supplementary material

Supplementary Figures
