# Supplementary material for: Suppression of autoimmune demyelinating disease by preferential stimulation of CNS-specific CD8 T cells using Listeria-encoded neuroantigen
Source: Sci Rep. 2017 May 8;7:1519. doi: 10.1038/s41598-017-01771-8 (PMC5431563; doi:10.1038/s41598-017-01771-8)

SUPPLEMENTARY MATERIALS

Suppression of autoimmune demyelinating disease by preferential stimulation of CNS-specific CD8 T cells using Listeria-encoded neuroantigen

Farah R. Itani[1,3], Sushmita Sinha[1], Ashley Brate[1,3], Lecia L. Pewe[2], Katherine N. Gibson-Corley[1], John T. Harty[1,2,3], and Nitin J. Karandikar[1,3,*]

From the [1]Department of Pathology, [2]Department of Microbiology, and the [3]Interdisciplinary Graduate Program in Immunology, University of Iowa, 200 Hawkins Dr., Iowa City, IA 52242

* Corresponding Author

Nitin Karandikar, MD, PhD
Department of Pathology,
University of Iowa Health Care
200 Hawkins Drive, C660 GH,
Iowa City, IA 52241
United States

E-mail address: nitin-karandikar@uiowa.edu

Supplementary Figure Legends:

Figure S1. Vector used to create recombinant *Listeria* expressing PLP175-194, adapted from *Lauer et. al*. The CNS peptide sequence was inserted between the *Bam*HI and *Pst*I restriction sites.

Figure S2. Peptide dose-response identifies PLP-specific CD8 T cells at various stimulatory concentrations. Intracellular cytokine staining was performed on bulk splenocytes harvested from LM-PLP infected B6 mice on d7 post infection. Splenocytes were left unstimulated, or stimulated with either OVA257-264 or varying concentrations of PLP178-191. Flow plots show antigen-specific IFN-γ production by CD8 (top panels) and CD4 (bottom panels) T cells.

Figure S3. LM-PLP infection of SJL/J mice leads to attenuation of EAE. Female 6-8 wk old SJL/J mice were infected with LM-PLP or LM-OVA on d-7 and immunized with PLP178-191/CFA on d0 and monitored for clinical disease. Data are representative of three independent experiments with at least seven mice per group per experiment. $^*p < 0.05$, $^{***}p < 0.001$.

Figure S4. Histological analysis of the spinal cord reveals less inflammation in LM-PLP infected animals immunized with PLP178-191/CFA.

H&E staining of lumbar spinal cord from infected and immunized B6 mice on d18 post-immunization depicts differences in inflammation (thick arrows) and axonal sheath swelling (thin arrows). Data are representative of at least two independent experiments.

Figure S1

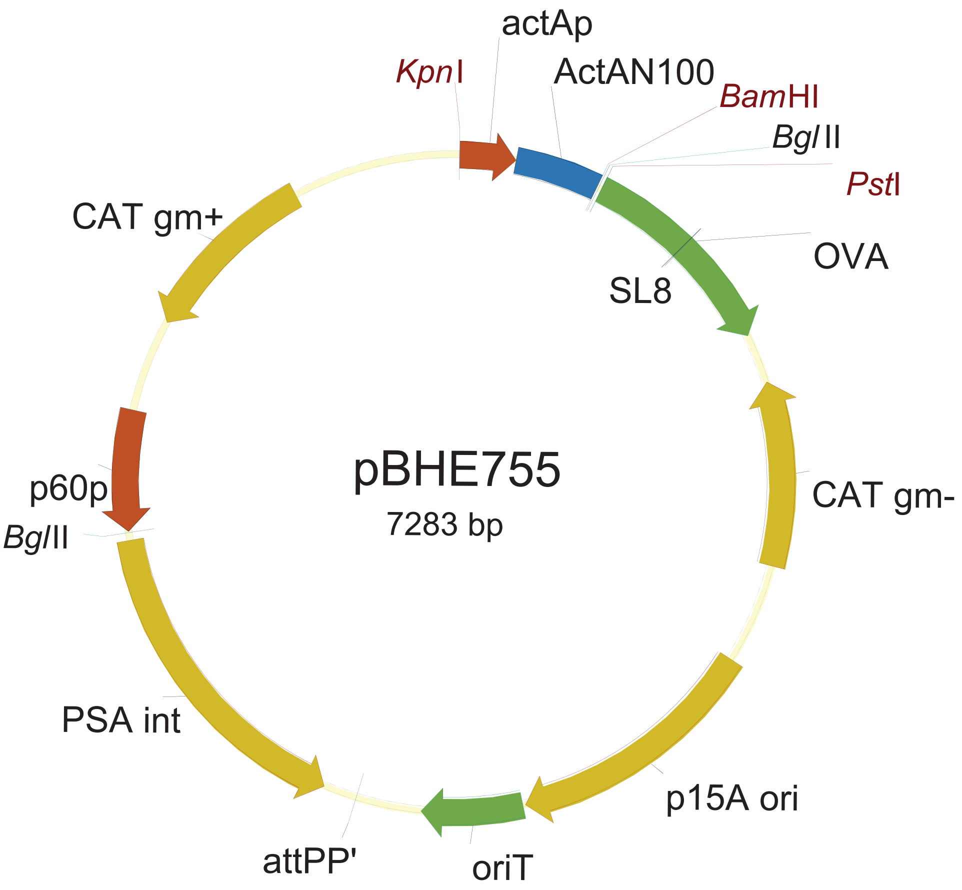
actAp
KpnI
ActAN100
BamHI
BglII
PstI
OVA
SL8
CAT gm+
pBHE755
7283 bp
p60p
BglII
CAT gm-
PSA int
p15A ori
attPP'
oriT

Figure S2

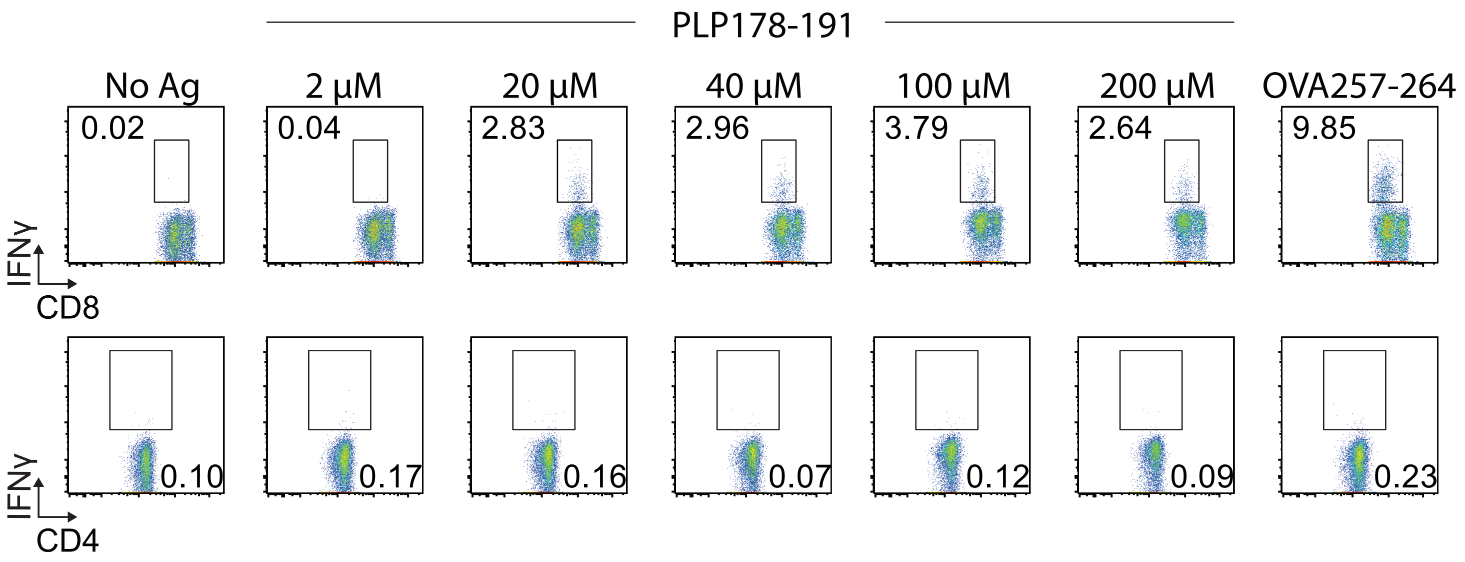
PLP178-191
No Ag
2 µM
20 µM
40 µM
100 µM
200 µM
OVA257-264
0.02
0.04
2.83
2.96
3.79
2.64
9.85
IFNγ
CD8
0.10
0.17
0.16
0.07
0.12
0.09
0.23
IFNγ
CD4

Figure S3

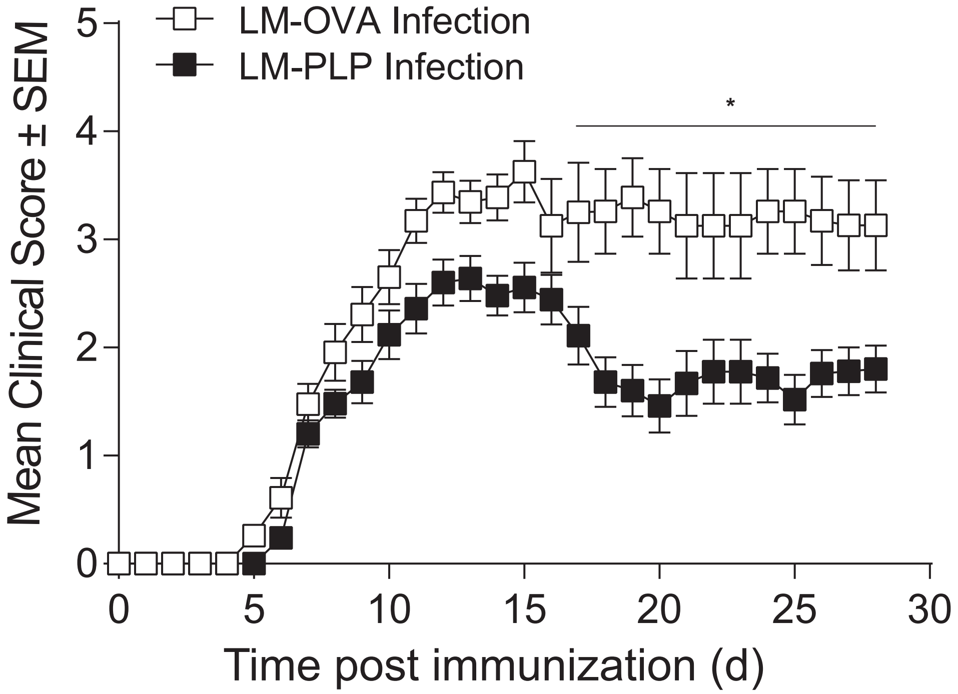

Infection	Onset	Peak***	Mortality***	CDI ***
LMOVA121-386	6.6 ± 1.2	4.2 ± 0.9	12/23	62.0 ± 22.9
LMPLP175-194	7.0 ± 0.8	3.24 ± 0.8	2/25	39.0 ± 16.4

Figure S4

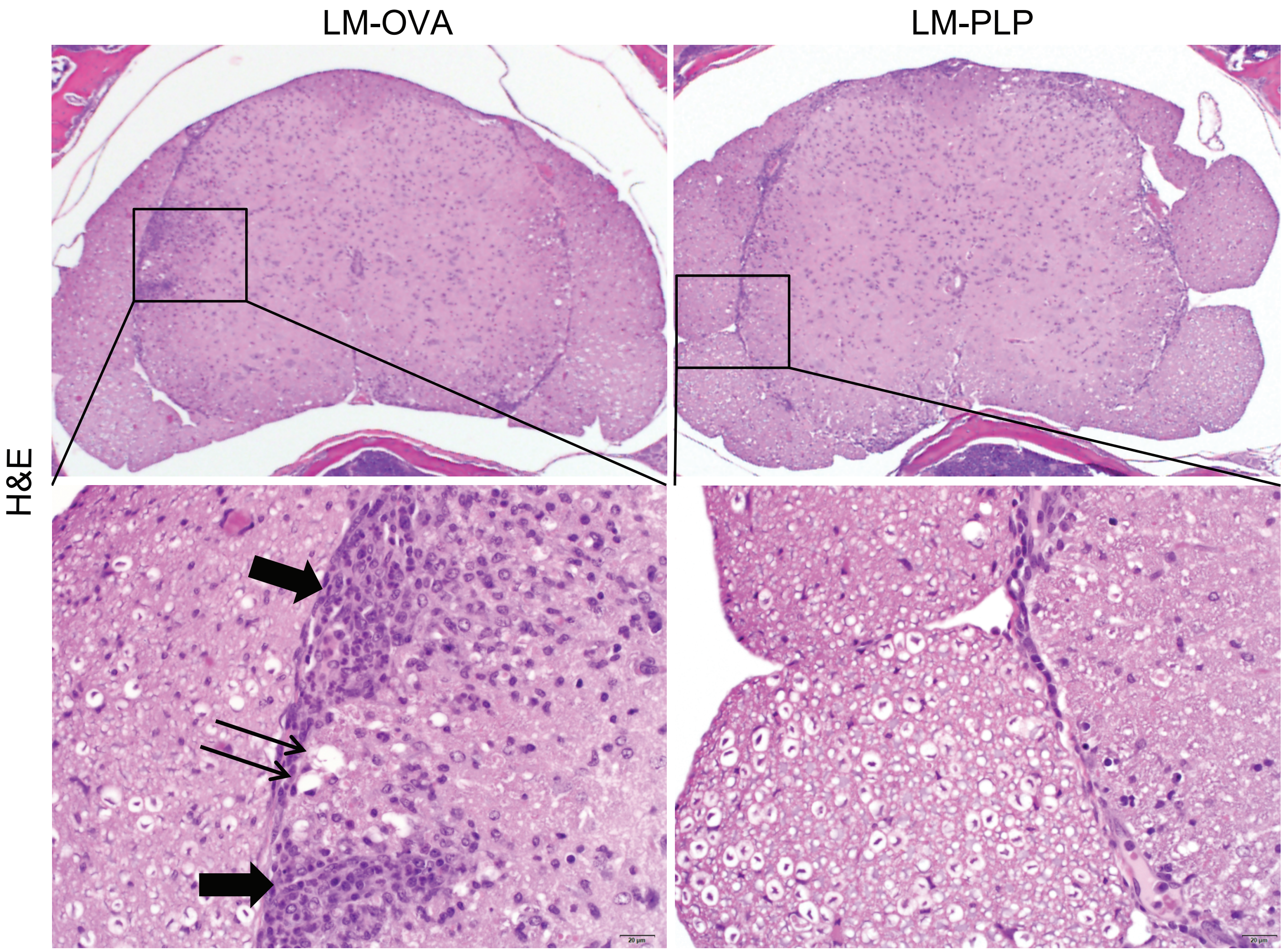

Supplement: Supplementary file 1 — Supplementary Figures [file 41598_2017_1771_MOESM1_ESM.pdf]
